# Effect of naive and cancer-educated fibroblasts on colon cancer cell circadian growth rhythm

**DOI:** 10.1038/s41419-020-2468-2

**Published:** 2020-04-27

**Authors:** Alessia Parascandolo, Raffaella Bonavita, Rosario Astaburuaga, Antonio Sciuto, Stefano Reggio, Enrica Barra, Francesco Corcione, Marco Salvatore, Gianluigi Mazzoccoli, Angela Relógio, Mikko O. Laukkanen

**Affiliations:** 1 Experimental Institute of Endocrinology and Oncology G. Salvatore, IEOS CNR, 80014 Naples, Italy; 2 Institute for Theoretical Biology (ITB), Charité—Universitätsmedizin Berlin, corporate member of Freie Universität Berlin, Humboldt-Universität zu Berlin and Berlin Institute of Health, 10117 Berlin, Germany; 3 Molekulares Krebsforschungszentrum (MKFZ), Charité—Universitätsmedizin Berlin, corporate member of Freie Universität Berlin, Humboldt-Universität zu Berlin and Berlin Institute of Health, 10117 Berlin, Germany; 4 Monaldi Hospital, 80131 Naples, Italy; 5 Dipartimento di Sanità pubblica, University of Naples Federico II, 80131 Naples, Italy; 6 IRCCS SDN, 80143 Naples, Italy; 7 Department of Medical Sciences, Division of Internal Medicine and Chronobiology Unit, IRCCS Foundation, Relief of Suffering, 71013 San Giovanni Rotondo, Italy; 8 Pineta Grande Research Laboratory, 81030 Caserta, Italy; 9 Present Address: Institute of Systems Medicine and Bioinformatics, Department of Human Medicine, MSH Medical School Hamburg—University of Applied Sciences and Medical University, Hamburg, 20457 Germany

**Keywords:** Cancer microenvironment, Cell growth, Circadian rhythms

## Abstract

Opportunistic modification of the tumour microenvironment by cancer cells enhances tumour expansion and consequently eliminates tumour suppressor components. We studied the effect of fibroblasts on the circadian rhythm of growth and protein expression in colon cancer HCT116 cells and found diminished oscillation in the proliferation of HCT116 cells co-cultured with naive fibroblasts, compared with those co-cultured with tumour-associated fibroblasts (TAFs) or those cultured alone, suggesting that TAFs may have lost or gained factors that regulate circadian phenotypes. Based on the fibroblast paracrine factor analysis, we tested IL6, which diminished HCT116 cell growth oscillation, inhibited early phase cell proliferation, increased early phase expression of the differentiation markers *CEA* and *CDX2*, and decreased early phase ERK5 phosphorylation. In conclusion, our data demonstrate how the cancer education of naive fibroblasts influences the circadian parameters of neighbouring cancer cells and highlights a putative role for IL6 as a novel candidate for preoperative treatments.

## Introduction

The biological clock regulates the expression level of proteins and their functional parameters, e.g., oscillation of cell growth, thereby coordinating cellular homeostasis and overall activity of the tissues. The disruption of the circadian rhythm has been proposed as a cause and/or a consequence of cancer^1^, suggesting a putative anti-tumorigenic role for normal circadian oscillation. However, the underlying mechanism explaining the correlation between markedly increased cancer risk and abnormal cellular circadian rhythm has remained elusive. Among the most important disrupted circadian parameters are the period of the oscillations, which indicates the length of one complete circadian cycle; the amplitude, which refers to one-half of the total predictable peak-to-trough change in the magnitude of the oscillation; and the acrophase^2–5^, which indicates the time of the first peak within the oscillation cycle. A plausible explanation for the lack of definitive mechanistic clarification of the disrupted circadian rhythm could be the multifaceted mutation-rich signal transduction networks in cancer cells that may enhance or suppress the cellular machinery that regulates the biological clock^6,7^.

Furthermore, the role of tumour stroma, another highly versatile environment, in the regulation of the circadian machinery in cancer cells is poorly understood. The coherent interaction between tumour stroma and cancer cells initiates epithelial cell immortalization as the gradually developing stroma begins to respond to the needs of the cancer cells^8^. The continuous interaction results in the development of a heterogeneous tumour stroma mosaic that is further affected by the status of the patient, e.g., the person’s age and pathological condition^8–12^. It has been recently and consistently demonstrated that the circadian clock regulates differentiation, cycling, and migration of mesenchymal stem cells and may thus contribute to the maintenance of the undifferentiated phenotype of these cells^13^. Additional insights into the role of the stroma was provided by a study showing that the microenvironment-coordinated mechano-chemical stiffness of normal breast epithelial cells may represent a risk factor for cancer development^14^.

We recently reported that the glycolytic activity of primary fibroblast is under the influence of the circadian clock machinery, demonstrating that time-dependent activity decreases in fibroblasts derived from normal tissues and there is more persistent glycolysis in tumour-associated fibroblasts (TAF)^15,16^. In the current work, we studied the effect of human colon adenocarcinoma-derived TAFs and their normal tissue counterparts, naive fibroblasts, on colon cancer growth oscillation and clock protein expression and analysed the effect of naive fibroblast-secreted IL6 on HCT116 cells. The data suggest prominent dampening of the HCT116 cell proliferation circadian rhythm resulting from the interaction with naive fibroblasts and by treatment with naive fibroblast secreted IL6. Conversely, TAFs led to increased growth oscillation in HCT116 cells. Based on the currently collected data, the tumour environment influences fibroblast paracrine factors that subsequently regulate the circadian rhythm of cancer cell growth.

## Materials and methods

### Primary fibroblast isolation

Normal colon naive fibroblasts and tumour-associated fibroblasts (TAFs) were derived from the same patient diagnosed with colon adenocarcinoma. The naive fibroblasts were isolated from the normal colon segment approximately 20 cm from the tumour close to the edge of the isolated colon segment that was histologically free of neoplasia. The isolated tissues were washed rigorously in phosphate-buffered saline (PBS), an antibiotic-antimycotic (Lonza, Basel, Switzerland), gentamycin (Lonza) and cut into pieces for incubation overnight at 37 °C in αMEM (Euroclone, Milano, IT) with antibiotic-antimycotic and gentamycin in the presence of collagenase type II 150 U/ml (Gibco, Waltham, MA, USA). The next day, the undigested tissue pieces were removed by centrifugation, and the supernatant was plated on gelatine (1 g/l) pre-treated dishes in αMEM (Euroclone) supplemented with 10% defined FBS (GE-Healthcare, Chicago, IL, USA), non-essential amino acids (Euroclone), L-alanine-L-glutamine (Euroclone), penicillin/streptomycin (Euroclone), antibiotic-antimycotic, and gentamycin.

### Primary fibroblast culture

The growth medium and antibiotics were replenished daily during the first three weeks in which the patient-derived fibroblasts were cultured. The first passage of the primary fibroblasts was performed after the cells were approximately 30% confluent in a dish in which several clones of the cells were grown. Primary human naive intestinal fibroblasts (HIFs) were purchased from Cliniscience (Cliniscience, Nanterre, France). The human colorectal cancer HCT116 and SW480 cell lines were purchased from ATCC, Manassas, VA, USA. All cells were grown in αMEM supplemented with 10% defined FBS, non-essential amino acids, L-alanine-L-glutamine (Euroclone), and penicillin/streptomycin. For the co-culture analysis, HCT116 and SW480 cells were seeded on coverslips (Waldemar Knittel Glasbearbeitungs-GmbH, Braunschweig, Germany) and let to grow to 40–50% confluence. The coverslips were transferred to the 70–80% confluent fibroblast cultures that were seeded three days earlier to allow paracrine factors to accumulate to the cell culture medium. Maximum 10 coverslips were plated to each fibroblast dish (100 × 20 mm). The cells kept in co-culture for 20 h, after which the coverslips were collected from the stromal cell dishes for the analysis.

IL6 and IL8 (both from Stem Cells, Vancouver, BC, Canada) were each added at 100 ng/ml to the HCT116 cell culture daily. For circadian rhythm analysis, the HCT116 cells seeded on the coverslips were allowed to grow on the fibroblasts for 24 h before the co-culture dishes were washed twice with serum free αMEM (Euroclone) and synchronized through incubation in 50% FBS (GE-Healthcare) for two h. The co-culture dishes were then washed twice with serum-free αMEM (Euroclone) and replenished with the αMEM and 10% defined FBS, non-essential amino acids, L-alanine-L-glutamine, and penicillin/streptomycin growth medium. The first analysis point was 20 h after the initiation of synchronized growth.

### Ethical permission

The ethical permission for the study was approved by IRCCS SDN (Comitato Etico per la Sperimentazione Clinica Progetto N:ro 2013-01-02) and Monaldi Hospital ethical committees (Deliberazione del Direttore Generale n:o 1239).

### DNA replication analysis

To analyse DNA replication, 10 mM bromodeoxyuridine (BrdU) (Roche, Basel, Switzerland) was added to the growth medium for a 15-minutes treatment. Subsequently, the cells were washed three times with PBS, fixed in 3% paraformaldehyde (Sigma, St. Louis, MO, USA) for 20 min at −20 °C, washed three times with PBS, and stained using anti-BrdU (Roche) primary antibody, FITC–conjugated secondary antibody (Jackson ImmunoResearch Laboratories Inc., West Grove, PA, USA), and Hoechst nuclear stain (Sigma). BrdU positive cells were counted from 30 microscope high power fields.

### Western blot analysis

The proteins were isolated from the cells homogenized in lysis buffer (50 mmol/L HEPES pH 7.5, 150 mmol/L NaCl, 10% glycerol, 1% Triton X-100, 1 mmol/L EGTA, 1.5 mmol/L MgCl_2_, 10 mmol/L NaF, 10 mmol/L sodium pyrophosphate, 1 mmol/L Na_3_VO_4_, 10 μg aprotinin/ml, and 10 μg leupeptin/ml) (Sigma). The following antibodies used: α-Clock (Santa Cruz, sc-25361, Dallas TX, USA), α-Bmal1 (Santa Cruz, sc-48790), and α-Per1 (Santa Cruz, sc-25362). α-Cry1 (Bethyl, A302-614A, Montgomery, TX, USA), α-TIMELESS (TIM) (Abcam, AB72458, Cambridge, UK), α-pERK1/2 (Thr202/Tyr204) (Cell Signaling, 4370S, Danvers, MA, USA), α-ERK1/2 (Cell Signaling, 9102S), α-cMYC (Santa Cruz, sc-40), α-cycD1 (Santa Cruz, sc-718 (M20)), α-CREB (Upstate, 06-863), α-pERK5 (Merck KGaA, 07-507, Darmstadt, Germania), α-ERK5 (Cell Signaling, 3552S), and α-tubulin (Cell Signaling).

### ProtoArray analysis

To analyse differences in cytokine and soluble receptor expression between naive fibroblasts and TAFs, we utilized Proteome Profiler kits (R&D Systems, Minneapolis, MN, USA) and human cytokine antibody arrays (Abcam). Whole-cell lysates were used for the array as instructed in the kits.

### Gene expression analysis

RNA was isolated using an RNeasy mini kit (Qiagen, Hilden, Germany). cDNA synthesis was performed by QuantiTect reverse transcription (Qiagen). SYBR green PCR master mix (Applied Biosystems, Foster City, CA, USA) was used for qPCR. The primers were human *CEA* forward catcgtgaaaccccatctct, human *CEA* reverse tctgttgccagactggagtg, human *CDX2* forward cccgaacagggacttgttta, human *CDX2* reverse agaccaacaacccaaacagc, human *FAP* forward tacgtttcatcactggccct and human *FAP* reverse catctgctgttccgtggatg, human Col1A1 forward gctactaccgggctgatgat and human *Col1A1* reverse accagtctccatgttgcaga, human *TENASCIN* forward acaacatcaagctgccagtg and human *TENASCIN* reverse ggggatgttgatgcgatgtg, human *Clock* forward aactgtgcctggactcttga, human *Clock* reverse cctaaagacactggctggga, human *Bmal1* forward ttgtcttcatccagccccat, human *Bmal1* reverse tatgttctggagcacgacgt, human *Per1* forward agcagacaacagcaaagacg, human *Per1* reverse caggtaggtgaggggaactg, human *Cry1* forward gacagccacatccaacttcc, human *Cry1* reverse cttcgtcaggagggttggat, human *TIM* forward gccatagccctcagtcatct, human *TIM* reverse ctgcctcttcaatcgtctgc, human *IL6* forward aggtgggcatggatttcaga, human *IL6* reverse gaaaggaggtgggtaggctt, human *IL6R* forward gaccgttcagcccgatatct, human *IL6R* reverse gaatcttgcagcctgatccg, human *IL8* forward gccagcttggaagtcatgtt, human *IL8* reverse ttcagagacagcagagcaca, human *IL8R* forward ggttgaggcagctatggaga, human *IL8R* reverse ggtcatctttgctgtcgtcc, human *18S RNA* forward gttggttttcggaactgagg, human *18S RNA* reverse gcatcgtttatggtcggaac, human *ACTIN* forward gctcgtagctcttctcca and human *ACTIN* reverse tgcgtgacattaaggaga. The samples were analysed in triplicates and qPCR reactions were repeated three times.

### Harmonic regression analysis

A harmonic regression analysis was used to determine the rhythmic characteristics of the gene and protein expression data. The R-package Harmonic Regression was used for the analysis^17^. The harmonic regression procedure fits a sine-cosine function, y(t) = m + a∙cos(wt) + b∙cos(wt), to the time-course data to estimate the best fitting amplitudes: A = √(a^2^ + b^2^) for a given period value. Periods from 18 h to 30 h were tested in increments of 0.1 h. For each condition, we reported the best fitting (lowest *p*-value) rhythmic parameters (period, amplitude, acrophase). The p-values were calculated with an F-test of model fit against a restricted model, mean-centring only (*p < 0.05, **p < 0.01, ***p < 0.001, ****p < 0.0001). The reported acrophase values correspond to the time of a peak within the experimental time range.

### Statistical analysis

The experiments were repeated at least three times. The *p*-values (*p < 0.05, **p < 0.01, ***p < 0.001) were determined by two-tail independent samples *t*-tests. The results are expressed as the mean ± SD.

## Results

### Naive colon fibroblasts alter the oscillation of cell proliferation and lead to a mild growth phenotype

Circadian rhythms have been studied extensively in cancer cells, which depict an altered oscillation phenotype and interconnected cellular functions that regulate growth, transformation, and metabolism^15,16,18–20^. Despite the importance of the cancer cell-educated tumour stroma in cancer progression, the function of stromal components in the regulation of circadian rhythms in cancer cells is poorly characterized. For this purpose, we isolated naive colon fibroblasts and their TAF counterparts (Supplementary Fig. 1a–d) from a patient with moderately differentiated intestinal adenocarcinoma that had infiltrated into the muscle layer and further to the subserosa connective tissue layer. The lymph nodes of the patient were positive, suggesting disease progression. A secondary observation revealed that the patient had cholecystitis.

**Fig. 1:**
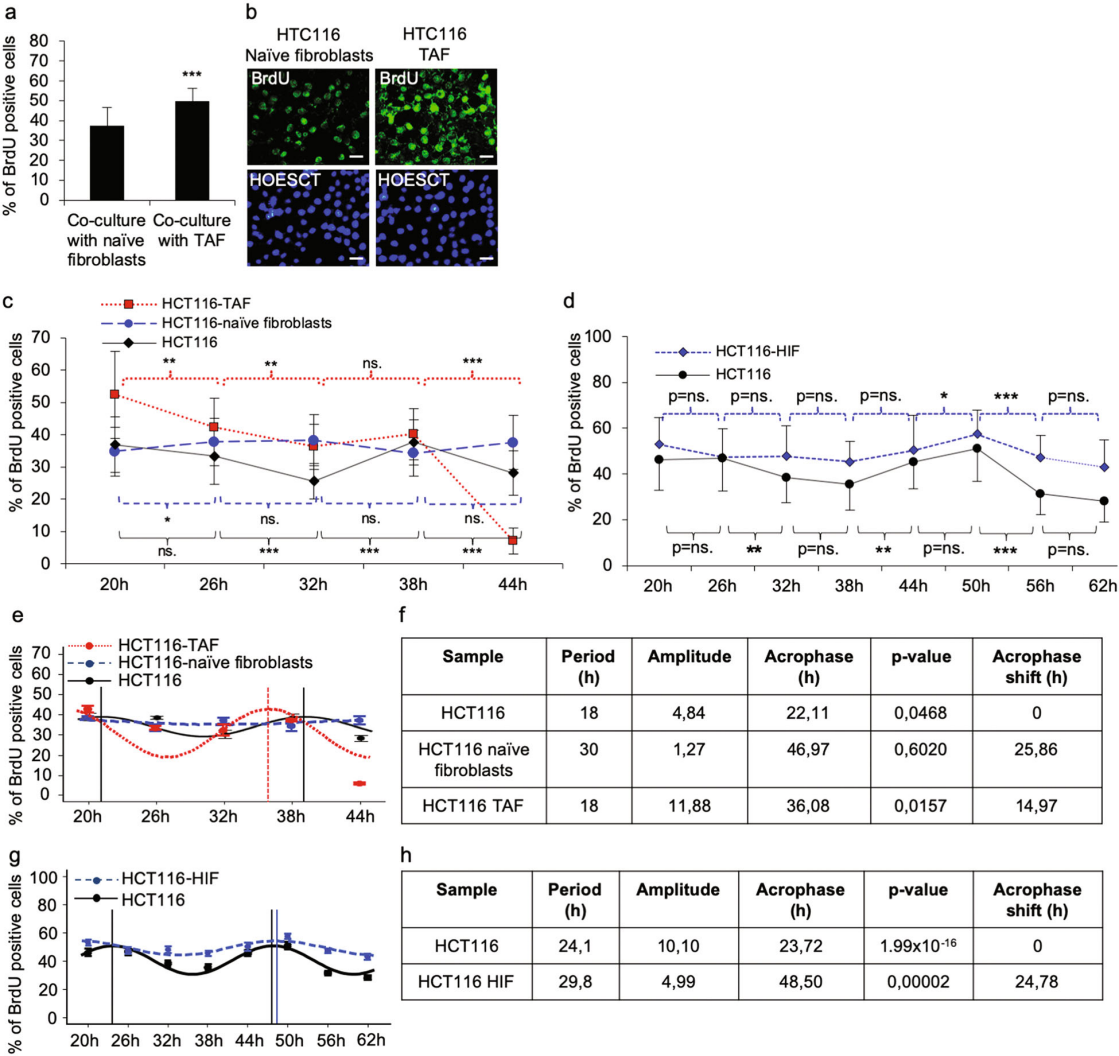
Effect of colon fibroblasts on colon cancer HCT116 cell DNA replication. **a**, **b** DNA replication analysis of the HCT116 cells grown on fibroblasts suggested increased proliferation for cancer cells grown together with TAFs. **c** Naive colon fibroblasts and colon TAFs modify the circadian rhythm of HCT116 cell DNA replication. The circadian rhythm of DNA replication in HCT116 cells decreased significantly (*p* < 0.001) between 26 h and 32 h, followed by a significant increase (*p* < 0.001) between 32 and 38 h, and a significant decrease (*p* < 0.001) between 38 and 44 h. HCT116 cells co-cultured with TAFs showed a similar circadian pattern in DNA replication as the HCT116 cells from the single-cell-type culture: a significant decrease between 20 and 26 h (*p* < 0.01) and between 26 and 32 (*p* < 0.01) followed by a moderate insignificant increase between 26 and 32 h and a significant decrease between 38 and 44 h (*p* < 0.001). The HCT116 cells co-cultured with naive colon fibroblasts demonstrated a dampened DNA replication circadian rhythm. **d** HIFs modified the circadian growth rhythm of HCT116 cell DNA replication, showing diminished circadian amplitude in the cells co-cultured with HIFs. **e** Harmonic regression analysis curve of HCT116 cells, HCT116 cells co-cultured with naive fibroblasts, and HCT116 cells co-cultured with TAFs. **f** Harmonic regression analysis parameters of HCT116 cells and HCT116 cells co-cultured with naive fibroblasts and HCT116 cells co-cultured with TAFs. The acrophase shift was calculated relative to the HCT116 cell control sample. **g** Harmonic regression analysis curve of HCT116 cells and HCT116 cells co-cultured with HIFs. **h** Harmonic regression analysis parameters of HCT116 cells and HCT116 cells co-cultured with HIFs. The acrophase shift was calculated relative to the HCT116 cell control sample. The *p*-values are *p < 0.05, **p < 0.01, and ***p < 0.001.

To characterize the tumour-supporting role of the TAFs, we performed a DNA replication assay showing significantly (*p* < 0.001) increased colon cancer HCT116 cell proliferation in the presence of TAFs compared to the HCT116 cells in co-culture with the normal colon naive fibroblasts (Fig. 1a, b). It has been well-established that cancer cells are able to promote fibroblast maturation to TAFs and further to cancer-associated fibroblasts (CAFs, myofibroblasts), which then promote the progression and expansion of cancer^21,22^. Therefore, in the current work, we analysed the circadian phenotype of the HCT116 cells in synchronized co-culture with primary naive fibroblasts isolated from normal colon and with TAF isolated from colon adenocarcinoma of the same patient. Strikingly, the results from the cell proliferation analysis conducted in 6-h intervals suggested significant differences in HCT116 cells cultured alone compared to those co-cultured with naive fibroblasts, which showed almost completely dampened growth oscillation. In contrast, the HCT116 cells co-cultured with TAFs showed enforced time-dependent differences in cell proliferation (Fig. 1c), corroborating the previously acquired data that suggested a growth-promoting role for TAFs^21,22^. The data suggesting milder cancer cell growth oscillation in the presence of naive fibroblasts was reinforced by analysis of the HCT116 cells co-cultured with human primary intestinal fibroblasts (HIFs), which model normal stroma, demonstrating similar attenuation of the circadian growth rhythm, as had been observed for the cells co-cultured in patient-derived naive fibroblast (Fig. 1d).

To analyse the modifications in the growth oscillation further, we performed a harmonic regression analysis, which confirmed that naive fibroblasts and TAFs induced significant alterations in the circadian parameters of the HCT116 cells (Fig. 1e, f). As demonstrated by DNA replication analysis, the period of the oscillation in cell proliferation was altered from 18 h for HCT116 cells in the single-cell-type culture to 30 h for those in the presence of naive fibroblasts, whereas it was unaffected in the cells co-cultured with TAFs (Fig. 1e, f). Interestingly, the acrophase, the time of the first peak the circadian curve, was delayed 25.86 h for the cells co-cultured with naive fibroblasts and 14.97 h for the cells co-cultured with TAFs. Additionally, the amplitude of the oscillation was reduced from 4.84 for the HCT116 cells control to 1.267 for the cells co-cultured with naive fibroblasts, and it increased to 11.88 for the cells co-cultured with TAFs (Fig. 1e, f).

Correspondingly, the harmonic regression analysis of the HCT116 cells co-cultured with primary HIFs culture corroborated the data obtained for the analysis of the patient-derived naive fibroblasts, demonstrating a lengthening of the period from 24.1 h for the cells in the HCT116 control condition to 29.8 h for the cells co-cultured with HIF (Fig. 1g, h) close to 30 h, as had been observed in the co-culture with patient-derived naive fibroblasts. Strikingly, the acrophase of the HCT116 cell growth oscillation, which was calculated to peak at 23.72 h, was delayed by 24.78 h and peaked at 48.50 h; these values are very similar to those obtained for the HCT116 cells in the naive fibroblast co-culture. Furthermore, as observed for the cells in the patient-derived naive fibroblast co-culture, the amplitude showed a reduction from the control HCT116 cell value of 10.10 to the HCT116 cell and primary HIF co-culture value of 4.99 (Fig. 1g, h).

### Naive fibroblasts and TAFs modify the oscillation of clock proteins and growth-related protein expression in HCT116 cells co-cultures

**Fig. 2:**
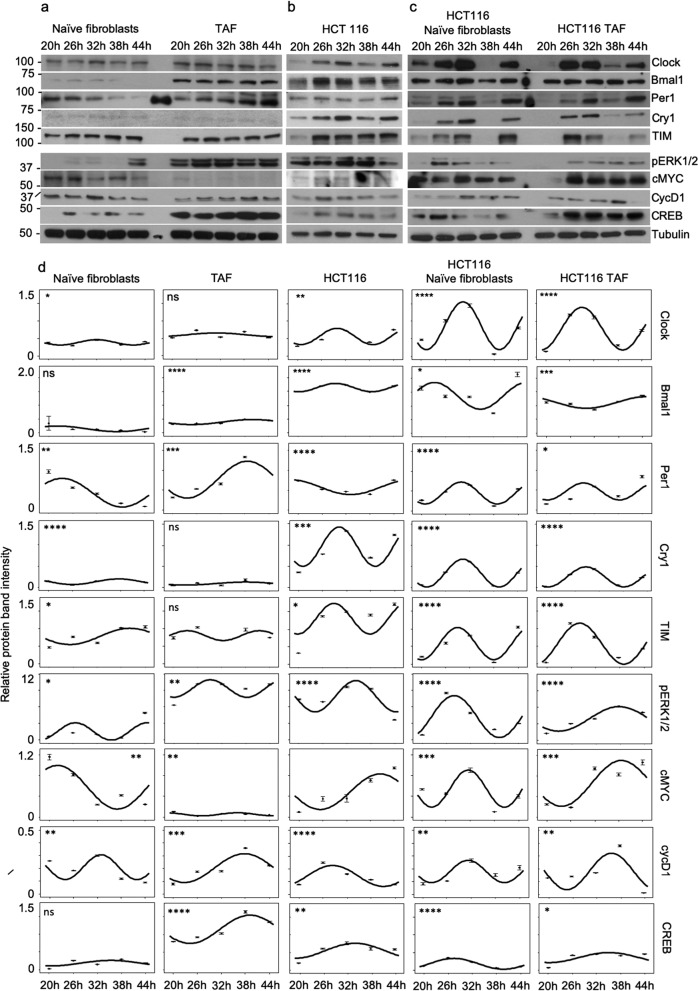
Western blot analysis of the expression levels of clock proteins and a panel of growth-related proteins. **a** Protein expression in naive fibroblasts and in TAFs. **b** Protein expression in HCT116 cells. **c** Protein expression in HCT116 cells co-cultured with naive fibroblasts and TAFs. **d** Harmonic regression analysis curves of clock proteins and growth-related molecules in naive fibroblasts, TAF, HCT116 cells, HCT116 cells co-cultured with naive fibroblasts, and HCT116 cells co-cultured with TAFs, according to western blot band intensities. The *p*-values are *p < 0.05, **p < 0.01, ***p < 0.001, and ****p < 0.0001.

Variation in the oscillation of clock gene expression has been suggested to increase cancer risk and to predict cancer progression and metastasis-free survival^23,24^. We studied the protein expression patterns of the clock genes Clock, Bmal1, Per1, Cry1, and TIM, and of the growth-related genes pERK1/2, cMYC, CycD1, and CREB involved in colon carcinogenesis. Interestingly, our data demonstrated that stromal fibroblasts and cancer cells cultured alone have a self-sufficient circadian rhythm in terms of protein expression, which did not show a prominent pattern, a finding that contradicts those of the DNA replication assay (Fig. 2a–d, Supplementary Fig. 2). The harmonic regression analysis of protein expression highlighted an aberrant modification in the circadian period and in acrophase that correlated to the differentiation of naive fibroblast to TAFs. The same pattern of modifications was not directly observed in the HCT116 cells in co-culture (Fig. 2d, Table 1). The expression analysis of *Clock*, *Bmal1*, *Per1*, *Cry1*, and *TIM* in SW480 colon cancer cells cultured alone or in co-culture with primary HIF corroborated the data described above, suggesting aberrant and self-sufficient expression pattern for the genes (Supplementary Fig. 3). These results support previous studies that reported altered circadian rhythm in mRNA and in protein expression correlating with carcinogenesis, further reinforcing the existence of a dysregulation of the circadian clock in cancer^25^.

**Table 1:** Harmonic regression analysis parameters.

Cells	Period (h)	Amplitude	Acrophase (h)	*p*-value	Acrophase shift (h)
Clock					
Naive fibroblasts	18	0.08	32.00	0.0116	2.16
TAF	30	0.05	30.99	0.3208	1.14
HCT116	18	0.23	29.85	0.0004	0
HCT116 naive fibroblasts	18.1	0.68	30.31	0.0000	0.47
HCT116 TAF	204	0.60	29.03	0.0000	−0.82
Bmal1					
Naive fibroblasts	30	0.10	22.03	0.4269	−7.38
TAF	30	0.09	39.82	0.0000	10.41
HCT116	18.2	0.15	29.41	0.0000	0
HCT116 naive fibroblasts	23	0.48	23.26	0.0133	−6.14
HCT116 TAF	30	0.21	44.97	0.0001	15.57
Per1					
Naive fibroblasts	30	0.36	23.34	0.0011	−24.80
TAF	30	0.64	40.61	0.0001	−7.53
HCT116	30	0.19	48.15	0.0000	0
HCT116 naive fibroblasts	18	0.27	29.84	0.0000	−18.31
HCT116 TAF	18	0.22	29.29	0.0451	−18.86
Cry1					
Naive fibroblasts	23.1	0.07	37.63	0.0000	7.47
TAF	30	0.04	38.18	0.1212	8.02
HCT116	18	0.46	30.16	0.0003	0
HCT116 naive fibroblasts	18.1	0.32	30.37	0.0000	0.21
HCT116 TAF	19	0.23	30.01	0.0000	−0.15
TIM					
Naive fibroblasts	30	0.24	40.17	0.0122	11.32
TAF	18	0.14	5.48	0.0956	−23.37
HCT116	18	0.43	28.85	0.0286	0
HCT116 naive fibroblasts	18	0.46	28.95	0.0000	0.10
HCT116 TAF	21.9	0.58	28.30	0.0000	−0.55
pERK1/2					
Naive fibroblasts	18	1.59	26.56	0.0116	−7.64
TAF	18.3	1.59	29.06	0.0042	−5.13
HCT116	20.3	2.83	34.20	0.0001	0
HCT116 naive fibroblasts	22.5	3.81	28.00	0.0000	−6.20
HCT116 TAF	30	2.19	38.04	0.0000	3.84
cMYC					
Naive fibroblasts	30	0.42	22.04	0.0012	−18.34
TAF	18	0.03	36.03	0.0023	−4.35
HCT116	30	0.34	40.38	0.0024	0
HCT116 naive fibroblasts	18	0.36	31.50	0.0007	−8.88
HCT116 TAF	30	0.44	38.63	0.0004	−1.75
cycD1					
Naive fibroblasts	18	0.10	32.90	0.0081	5.51
TAF	30	0.11	37.73	0.0003	9.34
HCT116	25.4	0.08	28.40	0.0001	0
HCT116 naive fibroblasts	18	0.09	31.23	0.0031	2.83
HCT116 TAF	22.3	0.14	35.94	0.0030	7.55
CREB					
Naive fibroblasts	30	0.08	35.40	0.0767	1.33
TAF	30	0.40	39.14	0.0000	5.07
HCT116	30	0.22	34.07	0.0023	0
HCT116 naive fibroblasts	24.6	0.15	27.47	0.0000	−6.60
HCT116 TAF	30	0.14	35.10	0.0227	1.02

Circadian period, amplitude, and acrophase, of naive fibroblasts, TAFs, HCT116 cells, HCT116 cells co-cultured with naive fibroblasts, and HCT116 cells co-cultured with TAFs. Parameters are based on the Western blot band intensities.

### Naive fibroblasts and TAFs have different patterns of paracrine factor expression

The results demonstrating altered DNA replication in the HCT116 cells co-cultured with TAFs, compared to that in HCT116 cells co-cultured with naive fibroblast, implied a loss or a gain of paracrine factors during differentiation of naive fibroblasts to TAFs, which might also contribute to the oscillation in cancer cell growth. Hence, we characterized the expression of paracrine factors from naive fibroblasts and TAFs and found large differences between the two cell populations. Based on the protein analysis, a number of growth-related proteins, extracellular matrix binding proteins and adhesion molecules, and cytokines and chemokines showed markedly different expression levels in the TAFs compared to the naive fibroblasts (Table 2, Supplementary Fig. 4, Supplementary Table 1). PDGF-BB, VEGF, FGF-9, TGF-β1 and TGF-β3, which are among the best well-characterized growth regulators, were upregulated, whereas the FLT-3 ligand, HER2, and HER4 were downregulated within the group analysed. The most prominently upregulated adhesion molecules were VCAM-1, ICAM-2, and integrin-β3, and the most prominently downregulated adhesion molecules were Cadherin-4, Cadherin-11, Cadherin-13, E-cadherin, and N-cadherin. Among the cytokines, IL-1α, INF-γ, SDF-1, and TNF-β were upregulated, whereas IL6, IL8, GM-CSF, and LIF were downregulated. The data reflected intricate phenotypic changes during naive fibroblast differentiation to TAFs that might be further influenced by the genotype of the cancer, type of cancer, and other unidentified factors related to the patient.

**Table 2:** Upregulated and downregulated paracrine factors in colon TAFs compared to naive fibroblasts.

	Upregulated	Downregulated
Growth related proteins	PDGF-BB	IGFBP-4
	SCF	IGFBF-3
	NT-4	Semaphorin 3°
	TGF-β1	TGF-β2
	TGF-β3	LRP-6
	IGFBP-2	HGF
	NT-3	FLT-3 LIGAND
	HB-EGF	CD147
	FGF-9	βIG-H3
	VEGF	ErbB4/HER4
	GDNF	BMPR-IB/ALK6
	PIGF	ErbB2/HER2
	Thrombopoietin	FGF-6
	ADAM9	
	CD138	
Adhesion molecules	Thrombospondin	Galectin-1
	Thrombospondin-2	CD66E
	Integrin β3	CD146
	Periostin	Cadherin-4
	VCAM-1	Cadherin-11
	ICAM-2	Cadherin-13
	NCAM-L1	E-Cadherin
	CD166	N-cadherin
	ADAM8	Integrin a9
	CD58	ESAM
	Nectin-4	ADAM10
Cytokines and chemokines	IL-1alfa	IL-5
	IL-12	IL-6
	IL-13	IL-7
	IL-15	IL-8
	IL-16	MCSF
	Eotaxin-3	MCP-1
	MIP-1B	GCP-2
	CX3CL1	EOTAXIN
	CXCR3	IP-10
	I-309	ENA-78
	PARC	LIF
	IFN-ɣ	Oncostatin M
	CCL5	GRO
	CCL13	GRO-alfa
	CCL22	GM-CSF
	CCL24	GCSF
	SDF-1	
	TNF-β	
	CK β8-1	
Others	CD90/Thy1	CD40
	Leptin	TIMP-2
	Galectin-3Bp	ECM-1
	HPRG	Coagulation factor II
	PAR1	
	NAP-2	
	MUCDHL	

The table lists proteins that were at least 1.5-fold upregulated and at least 0.25-fold downregulated.

### IL6 promoted short-term growth inhibition and early phase differentiation of HCT116 cells

**Fig. 3:**
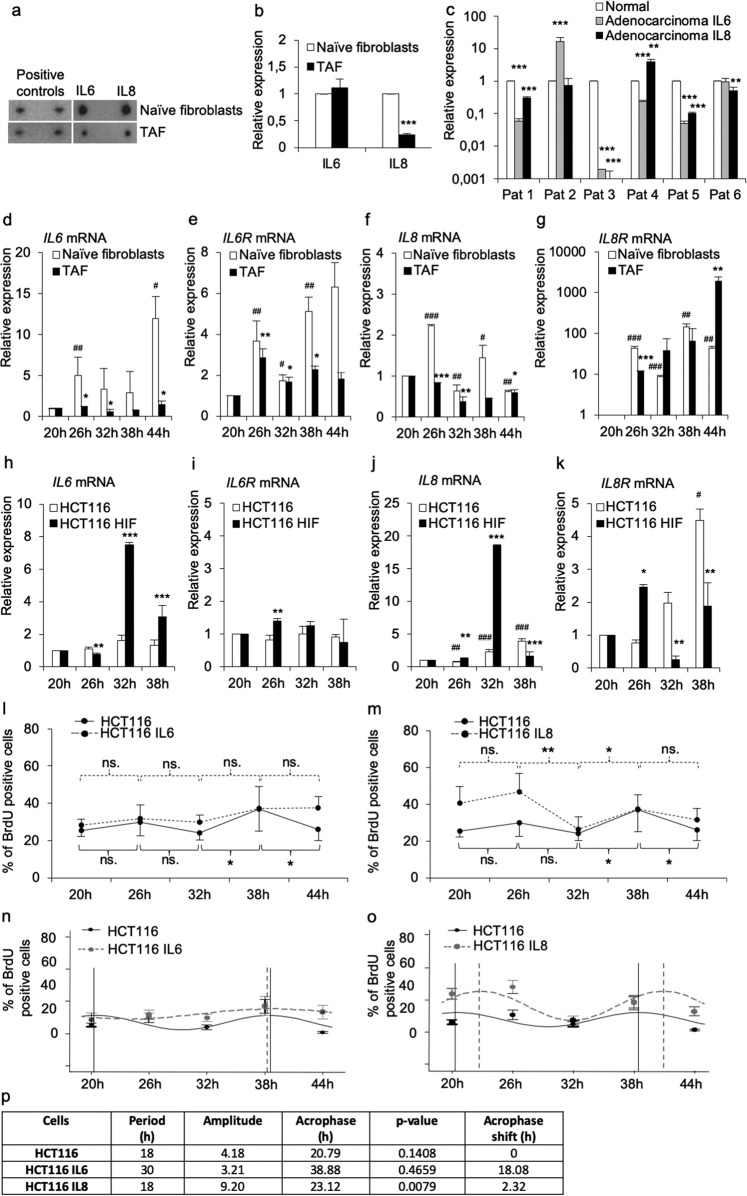
Circadian rhythm growth phenotype of HCT116 cells treated with IL6 and IL8. **a** ProtoArray data suggesting downregulated IL6 and IL8 expression in TAFs compared to their expression in naive fibroblasts. Positive control dot intensities are shown on the left. **b** The mRNA expression levels of *IL6* and *IL8* in naive fibroblasts and in TAFs. **c** The mRNA expression analysis of *IL6* and *IL8* from normal colon, benign adenomas, and adenocarcinomas isolated from the same patient. **d** The mRNA expression analysis of *IL6* in naive fibroblasts and in TAF. **e** The mRNA expression analysis of *IL6R* in naive fibroblasts and in TAF. **f** The mRNA expression analysis of *IL8* in naive fibroblasts and in TAF. **g** The mRNA expression analysis of *IL8R* in naive fibroblasts and in TAF. **h** The mRNA expression analysis of *IL6* in HCT116 cells cultured alone and at the presence of HIF. **i** The mRNA expression analysis of *IL6R* in HCT116 cells cultured alone and at the presence of HIF. **j** The mRNA expression analysis of *IL8* in HCT116 cells cultured alone and at the presence of HIF. **k** The mRNA expression analysis of *IL8R* in HCT116 cells cultured alone and at the presence of HIF. **l** DNA replication analysis of the HCT116 cells treated with IL6 demonstrated dampened circadian rhythm amplitude, suggesting a lack of significant differences in cell proliferation during the 44-h follow-up, whereas the growth for the control HCT116 cells was significantly different at time points between 32 and 38 h (*p* < 0.05) and between 38 and 44 h (*p* < 0.05). **m** The incubation of HCT116 cells with IL8 significantly enhanced time-dependent DNA replication during the 20–44 h follow-up period. **n** Harmonic regressio**n** analysis curve of the HCT116 control cells and the IL6-treated cells. The analysis is based on the DNA replication results. **o** Harmonic regression analysis curve of the HCT116 control cells and the IL8-treated cells. The analysis is based on the DNA replication results. **p** Harmonic regression analysis parameters period, amplitude, and acrophase for the IL6 and IL8 treatments. The *p*-values are *p < 0.05, **p < 0.01, and ***p < 0.001.

Next, we investigated whether the downregulation of paracrine factors in TAFs could explain the differences in DNA replication and protein expression observed in HCT116 cells co-cultured with naive fibroblasts and those co-cultured with TAFs. The effect of growth factors and adhesion molecules is well-characterized in tumorigenesis, whereas the function of certain cytokines, such as IL6 and IL8, both downregulated in TAFs, has received less attention. Notably, the IL6 and IL8 cytokine expression was downregulated in TAFs as compared to naive fibroblasts (Fig. 3a) that was further corroborated by the expression analysis from the cellular mRNA and from whole tumour samples supporting their frequent downregulation in adenocarcinomas (Fig. 3b, c). This finding was in line with previous reports showing a correlation between increased IL6 expression, progression of tumorigenesis, and cellular differentiation^26–28^. The time-dependent mRNA expression analysis of *IL6* and *IL6 receptor* (*IL6R*) in naive fibroblasts and in TAFs further reinforced the data demonstrating decreased gene expression of *IL6* in TAFs (Fig. 3d) that coincided with decreased expression of *IL6R* (Fig. 3e). The time-dependent expression analysis of *IL8* and *IL8 receptor* (*IL8R*) suggested similar downregulation of the cytokine (Fig. 3f), although the *IL8R* mRNA expression showed aberrant expression pattern (Fig. 3g). The time-dependent mRNA expression of *IL6*, *IL6R*, *IL8* and *IL8R* in HCT116 cells that were cultured alone or in the presence of primary HIF suggested an increased tendency of the cytokine and of the cytokine receptor expression in the co-culture (Fig. 3h–k). The expression studies of the cytokines and their receptors in SW480 cells grown alone or in the presence of primary HIF corroborated the observed increased expression tendency of *IL6* in SW480 cells in the presence of primary HIF (Supplementary Fig. 5a) and a more random expression pattern for *IL6R*, *IL8* and *IL8R* in the co-culture (Supplementary Fig. 5b–d).

We, therefore, analysed the effect of IL6 and IL8 supplementation in the proliferation of HCT116 cells in single cell culture. Interestingly, IL6 supplementation diminished the oscillation of DNA replication in the HCT116 cells showing lack of statistical differences between time points (Fig. 3l). Conversely, IL8 supplementation of the HCT116 cells was associated with increased oscillation of cell proliferation (Fig. 3m).

Results from the harmonic regression analysis of IL6 treatment demonstrated markedly similar circadian phenotype values for DNA replication (Fig. 3n, p) as those observed in the co-culture of the HCT116 cells with naive fibroblasts. The period of DNA replication for the HCT116 cells was 18 h (Fig. 3p), as observed for the cells co-cultured with naive fibroblasts (Fig. 1f). The amplitude decreased from 4.18 to 3.21, which was more moderate than that observed for the cells co-cultured with naive fibroblasts, and the acrophase was delayed, as had been observed for the cells co-cultured with the naive fibroblasts, from 20.79 to 38.88 h, demonstrating an 18.08 h shift in growth oscillation (Fig. 3n, p). Therefore, the IL6 expression that was lost in the course of naive fibroblast differentiation to TAFs may contribute to a mild circadian cell proliferation phenotype. The harmonic regression analysis for the cells supplemented with IL8 showed no change in the period of cell proliferation, but instead demonstrated increased amplitude values, from 4.18 to 9.20, and a delay in the acrophase by 2.32 h (Fig. 3o, p). Although IL8 was downregulated in TAFs, the supplementation of IL8 to the HCT116 cell culture led to growth modifications that were similar to those of the TAFs, suggesting that the expression of tumour suppressors and tumour promoters is modified during fibroblast differentiation.

### IL6 and IL8 treatment of HCT116 cells affect time-dependent clock protein expression and ERK1/2 phosphorylation

**Fig. 4:**
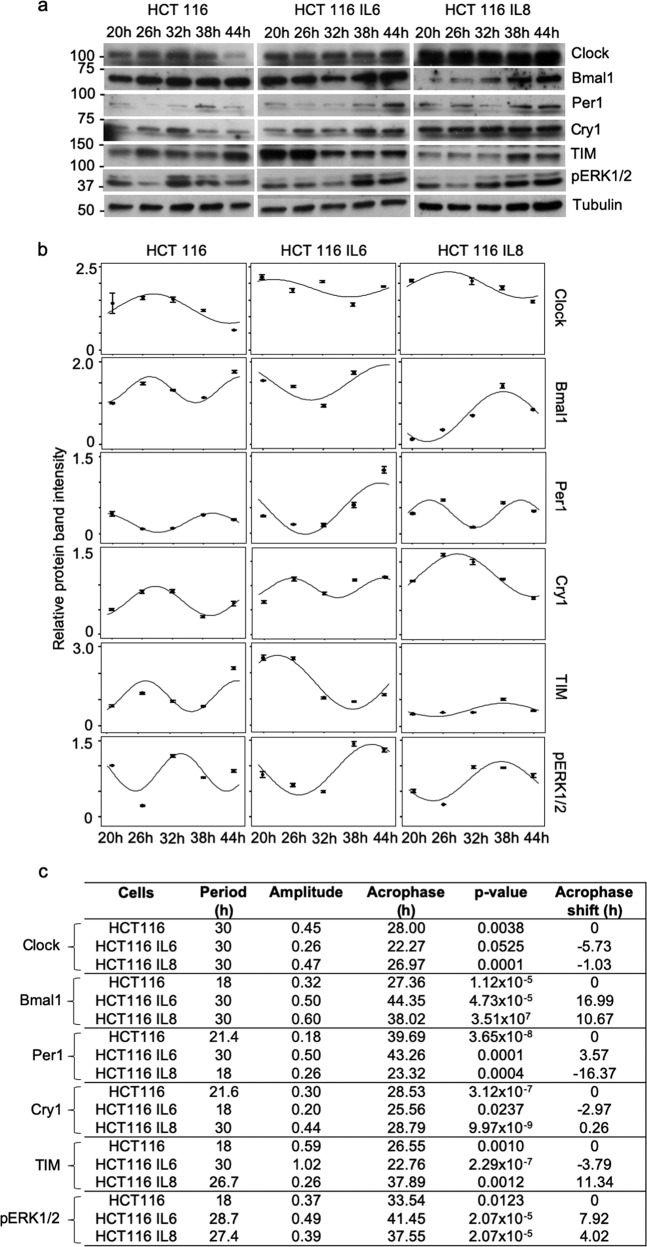
Circadian rhythm protein expression phenotype of HCT116 cells treated with IL6 and IL8. **a** Results from the time-dependent western blot analysis of protein expression in the control HCT116 cells, HCT116 cells treated with IL6, and HCT116 cells treated with IL8. **b** Harmonic regression analysis curves based on the western blot band intensities. **c** Harmonic regression analysis of the circadian parameters based on the western blot band intensities.

The results from the Western blot analysis of IL6 and IL8 supplementation to the HCT116 cell culture revealed alterations in Clock, Bmal1, Per1, Cry1, TIM, and pERK1/2 protein activation levels compared to the levels found for the untreated controls (Fig. 4a, Supplementary Fig. 6). Similarly, as observed in the HCT116 cells co-cultured with the fibroblasts (Fig. 2), there was no specific expression pattern that could be characterized as an increase or decrease in cell aggression, as was implied by the results of the harmonic regression analysis (Fig. 4b, c). The circadian parameters (period, amplitude, and acrophase) were all modified by IL6 and IL8 supplementation, but the response varied for each clock protein. The data confirmed that the protein expression did not show a significant oscillatory behaviour, whereas the functional response, i.e., DNA replication, had a particular recognizable pattern in growth oscillation that corresponded to the type of stimulus.

### IL6 has early and late effects on HCT116 cell growth

**Fig. 5:**
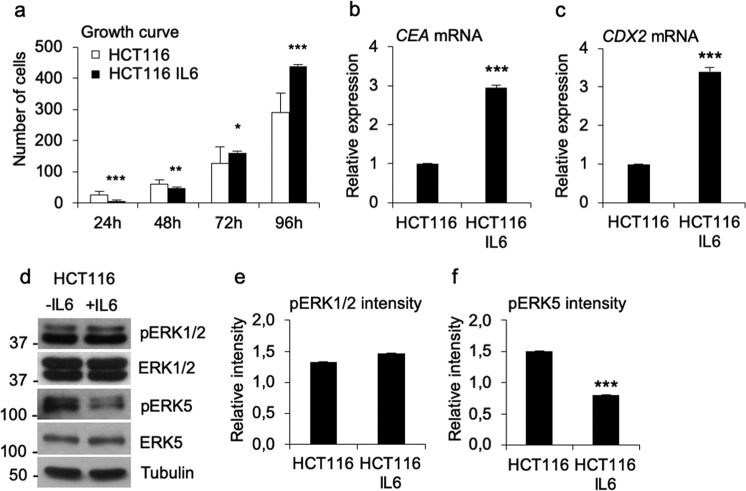
IL6 has early and late effects on HCT116 cell growth. **a** The growth curve of IL6-treated HCT116 cells shows significant growth inhibition at the early phase 24 h (*p* < 0.001) and 48 h (*p* < 0.01) time points and significantly increased cell numbers at the 72 h (*p* < 0.05) and 96 h (*p* < 0.001) time points. **b** The results from the mRNA analysis of the differentiation marker *CEA* in the IL6-treated HCT116 cells show significantly (*p* < 0.001) increased expression. **c** The differentiation marker *CDX2* mRNA analysis in the IL6-treated HCT116 cells shows significantly (*p* < 0.001) increased expression. **d** The results from the Western blot analysis of ERK1/2 and ERK5 phosphorylation in IL6-treated HCT116 cells demonstrated no differences in ERK1/2 activation status but significantly decreased ERK5 phosphorylation. **e**, **f**. Intensity analysis of ERK1/2 and ERK5 phosphorylation. The *p*-values are *p < 0.05, **p < 0.01, and ***p < 0.001.

Because the altered growth oscillation caused by IL6 treatment suggested reduced HCT116 cell aggressiveness, we performed a growth curve analysis that showed significantly reduced HCT116 cell numbers during the first 48 h of treatment. Unexpectedly, the IL6-derived initial growth suppression was followed by enhanced growth at the 72-h and 96-h time points, pointing to early and late phase effects on cell proliferation (Fig. 5a). To further dissect the effect of IL6 on HCT116 cells and to analyse the reduced cellular growth in the early phase of treatment, we studied the expression of differentiation markers 48 h after initiating the IL6 treatment. The results showed significantly (*p* < 0.001) increased *CEA* and *CDX2* mRNA expression (Fig. 5b, c), suggesting that IL6 promoted HCT116 cell differentiation as an immediate early effect, which could further affect the frequency of cell cycling. Recent works have indicated the importance of ERK5 kinase in the progression of colon carcinoma even in tumours with downregulated ERK1/2 phosphorylation^29,30^. Inspired by this finding, we analysed ERK1/2 and ERK5 phosphorylation by Western blot analysis and found a significant (*p* < 0.001) IL6 treatment-derived reduction in ERK5 phosphorylation at the 48 h time point. Although the ERK1/2 activation level remained the same, the reduced growth stimulation may have been the result of ERK5 inactivation, which would align with the impact of increased cell differentiation (Fig. 5d–f). Hence, the reduced ERK5 kinase activation together with the increased differentiation may contribute to the growth inhibition observed during the early-phase of IL6 treatment (48-h timepoint) and to the dampened circadian rhythm of the HCT116 cells in the presence of naive fibroblasts observed at the 44-h follow-up period.

## Discussion

Because of their marked diversity, tumours were originally characterized as organ-like structures not under the normal biological growth control^8,31^. Another early study, published in 1912 by Isaac Levin, suggested that “cells of a normal organ are capable of inhibiting tumour growth, whereas impairment of the normal state of the organ is crucial for the growth of the tumour”^32^. Although cancer has long been considered a multifactorial disease, recent discoveries have provided greater and more diverse insight into this pathological condition, which subjugates normal cells to protect and promote cancer progression^33,34^. Since cancer was first described, the gradual understanding of the interplay between cancer cells and tumour stroma has exposed sophisticated liaisons that serve the needs of each region of the mosaic tumour^11,12^. More importantly, various model systems used to study tumour function have provided more comprehensive results that show the paracrine effect of the different stromal components influencing the cancer cell biology, which otherwise is coordinated by the overexpression of single oncogenes^35^.

In the current work, we studied the circadian rhythm of cancer cell growth and protein expression in a model in which the oscillation of both the HCT116 and SW480 cancer cells and patient-derived stromal fibroblasts were synchronized. The data suggested a milder growth oscillation for the HCT116 cells in the presence of naive colon fibroblasts, whereas the presence of TAFs led to a significantly enhanced effect. The altered proliferation was observed in conjunction with the modification of time-dependent clock protein and activation of growth-related signalling molecules. Importantly, the results demonstrated a reproducible pattern in the oscillation of HCT116 cellular proliferation, whereas the aberrant mRNA and protein activation lacked a characteristic circadian phenotype. To date, the literature has reported an abnormal oscillation of protein expression in carcinogenesis^23,24^, but the effect of patient-derived fibroblasts on the circadian growth pattern of cancer cells is poorly characterized. Therefore, our results provide reference values that characterize the circumstances in which cancer cells interact with neighbouring fibroblasts, which may enable comparisons of the interactions before and after cancer cells have educated naive fibroblasts to promote tumorigenesis. Cancer cell education of stromal components is elementary to cancer progression^36–41^ and may be a programmed mechanism in cancer cells that suppresses autocrine functions^42–44^ that are not directly related to malignancy. The phenotypic changes that occur as naive fibroblasts differentiate to TAFs and the subsequent differentiation to cancer-associated fibroblasts (CAFs, myofibroblasts) is also linked to increased and decreased expression levels of paracrine factors, which are among the most prominent stromal modifications. TAF and CAF paracrine factors provide cancer cells with an appropriate microenvironment for nurturing tumour growth and expansion^45,46^. Additional support for the importance of paracrine modifications was provided by a recent work that demonstrated the marked influence of adhesion molecules on the regulation of core-clock protein expression^14^ and was corroborated by our data showing markedly aberrant expression of cadherin family members in TAFs. Moreover, downregulation of cytokines in naive fibroblast constitutes another interesting area of study, as this group of proteins may contain factors with growth-limiting effects on tumours^47,48^, such as that shown by IL6 in the current work. IL6 supplementation mimicked the effect of naive colon fibroblasts on oscillation of HCT116 cellular proliferation during the 44-h follow-up period and reduced cancer cell growth, which, based on our current data, was presumably caused by reduced ERK5 phosphorylation and increased differentiation of cancer cells. Notably, the immediate short-term growth inhibitory effect of IL6 supplementation may inspire a novel case-dependent preoperative drug regimen candidate that is likely to reduce the aggressiveness of colon adenocarcinoma.

In conclusion, the analysis of the circadian rhythm of colon cancer cells in synchronized co-culture with naive fibroblasts or with TAFs showed significantly altered growth oscillation phenotypes. Our proof-of-principle work provides novel insights into a putative mechanism how naive fibroblasts regulate cancer cell growth. Interestingly, the altered growth oscillation, which differed from the dysregulated oscillation in protein expression, suggested a specific pattern correlating to the fibroblast-derived stimulus of cancer cells. The lack of a defined pattern in the circadian rhythm of protein expression could be due to the highly diverse regulation of gene expression, which is influenced by mutations^49^, epigenetic factors^50^, and stromal components^35^. Although the same factors influence the growth and proliferation indexes of tumour cells, as a function of cancer cells, growth is a singular event that differs in complexity from the protein synthesis, which could explain the observed data.

## Supplementary information

Supplementary Figure 1

Supplementary Figure 2.

Supplementary Figure 3.

Supplementary Figure 4.

Supplementary Figure 5.

Supplementary Figure 6.

Supplementary Table 1.

SUPPLEMENTARY FIGURE AND TABLE LEGENDS
