# Supplementary figures and images for: Effect of naive and cancer-educated fibroblasts on colon cancer cell circadian growth rhythm
Source: Cell Death Dis. 2020 Apr 27;11(4):289. doi: 10.1038/s41419-020-2468-2 (PMC7184765; doi:10.1038/s41419-020-2468-2)

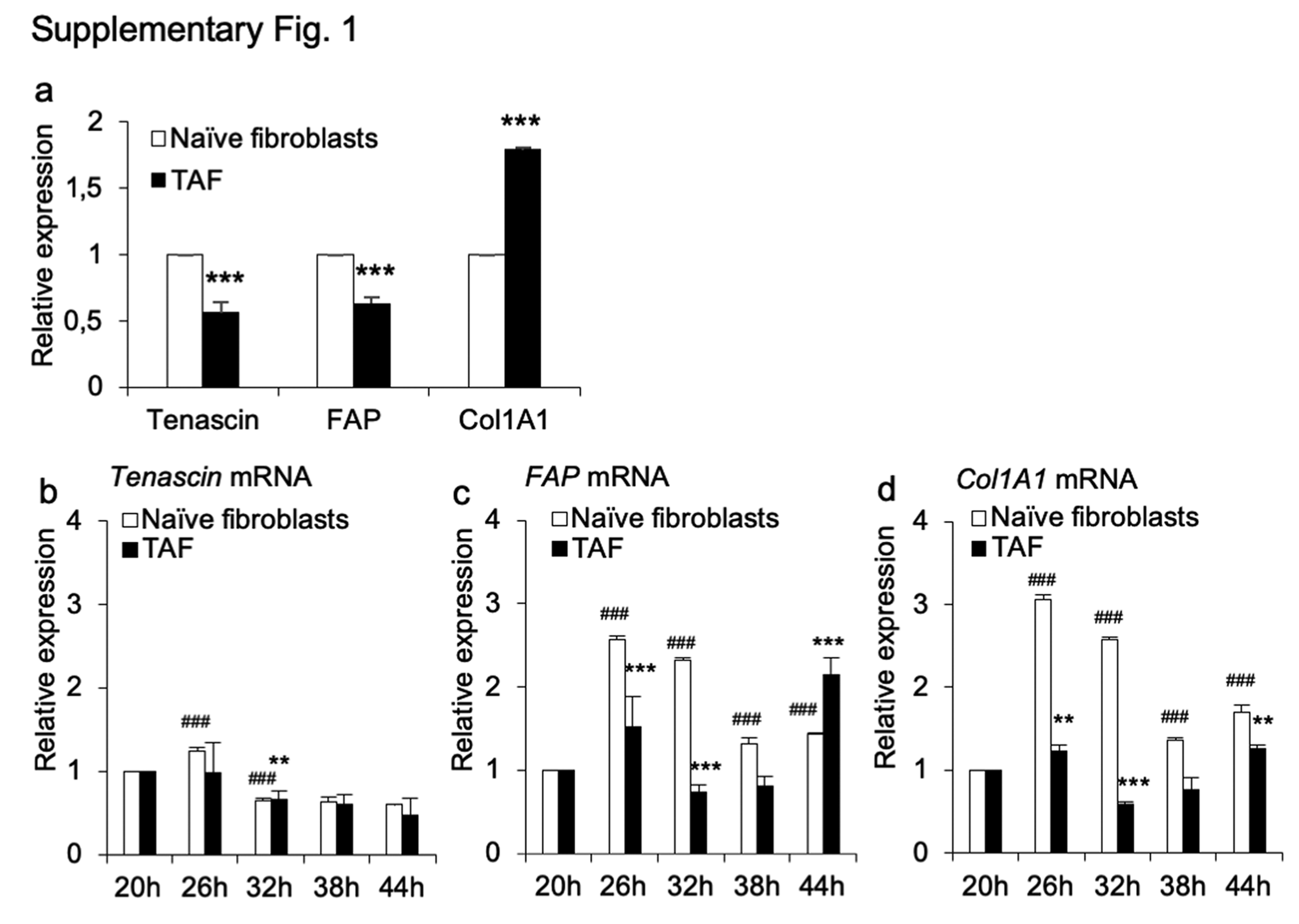

Supplement: Supplementary file 1 — Supplementary Figure 1 [file 41419_2020_2468_MOESM1_ESM.tif]

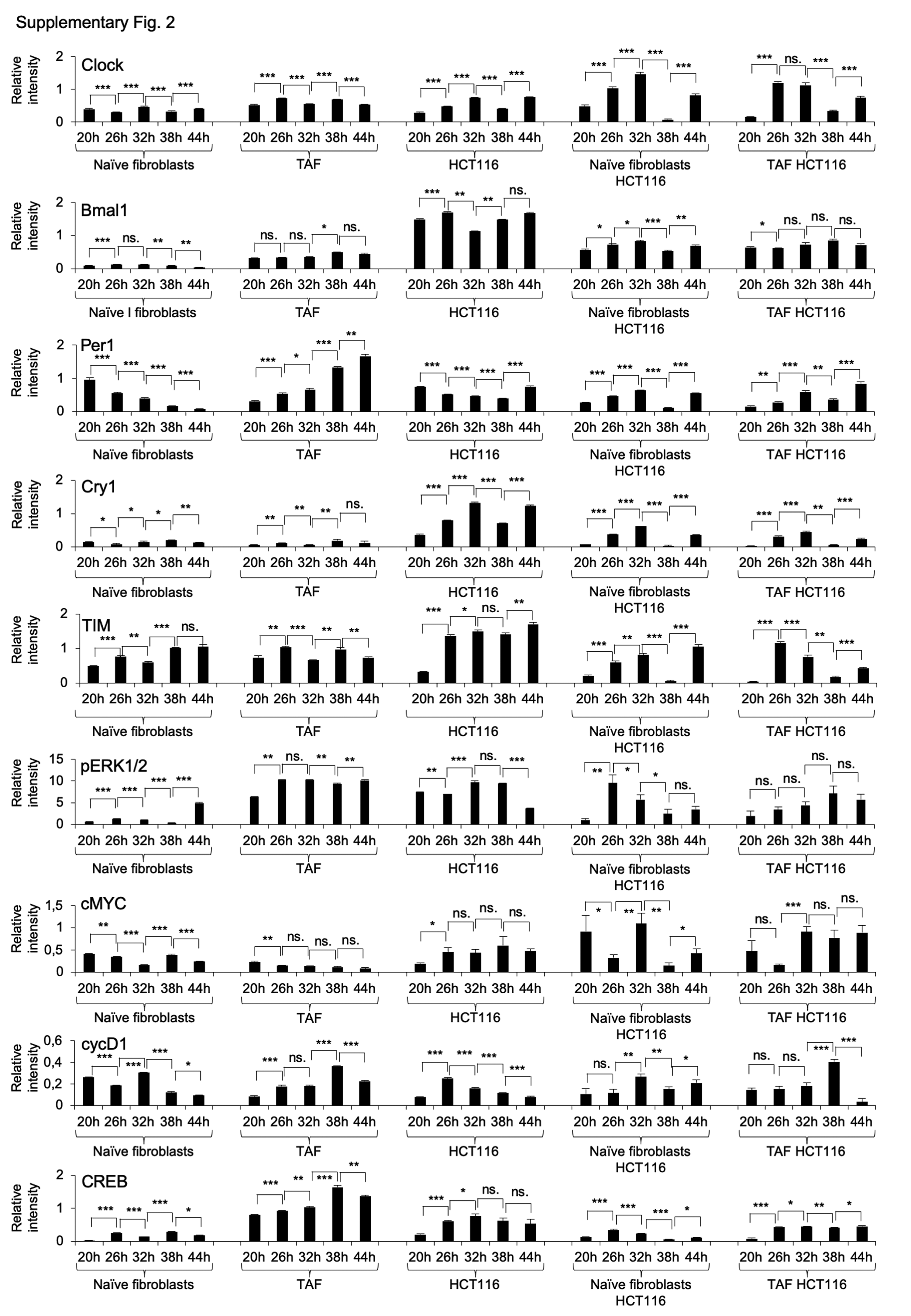

Supplement: Supplementary file 2 — Supplementary Figure 2. [file 41419_2020_2468_MOESM2_ESM.tif]

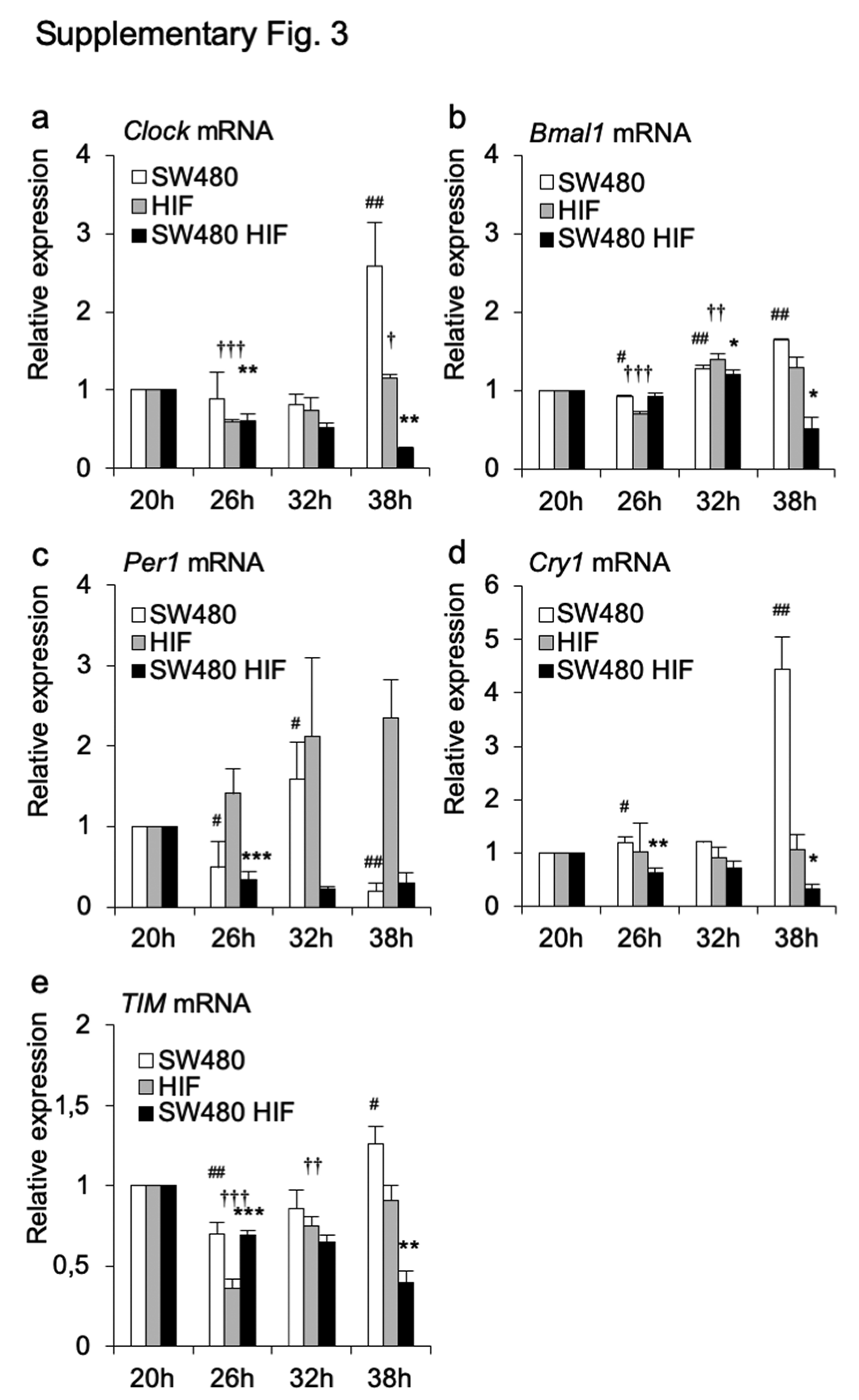

Supplement: Supplementary file 3 — Supplementary Figure 3. [file 41419_2020_2468_MOESM3_ESM.tif]

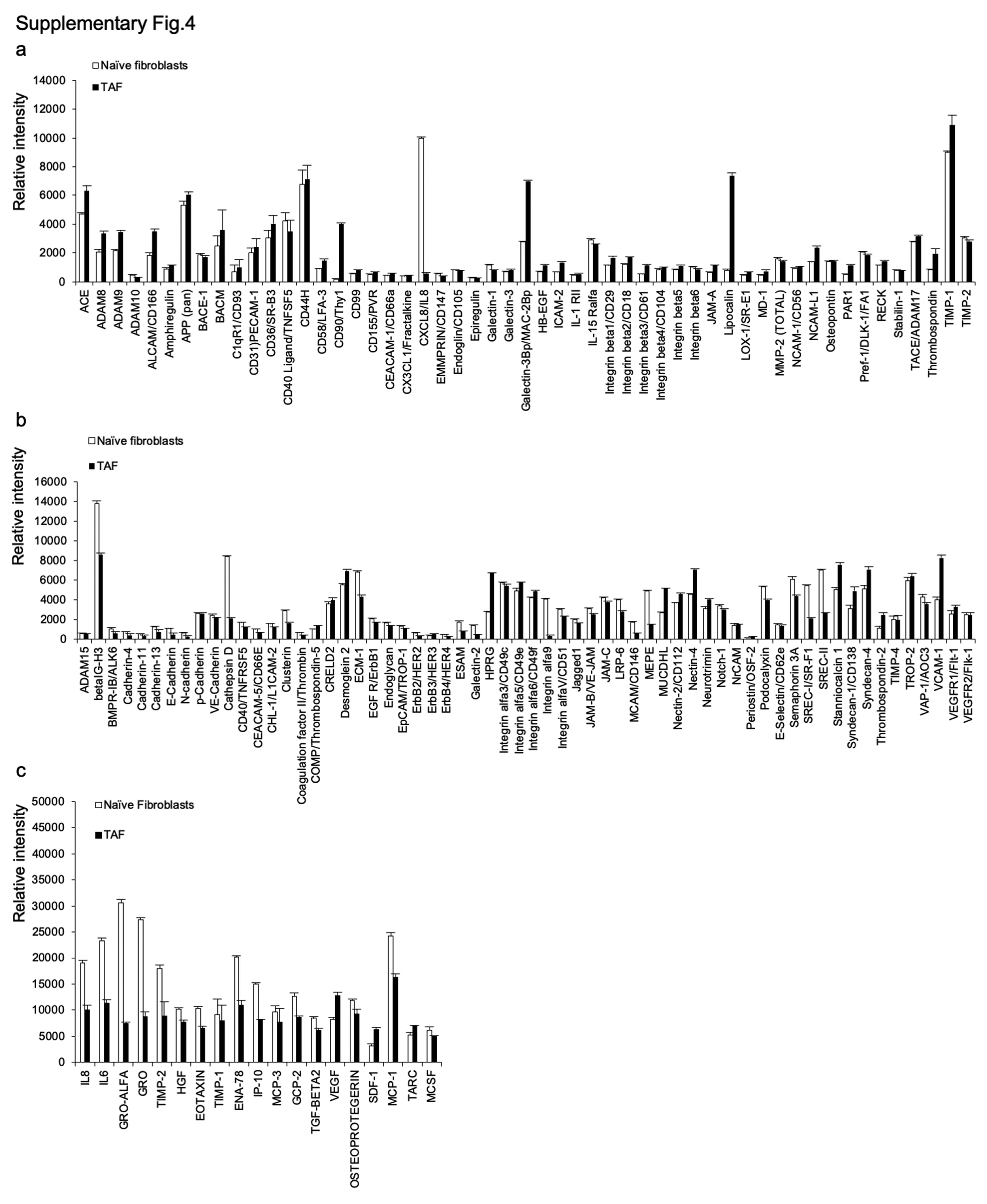

Supplement: Supplementary file 4 — Supplementary Figure 4. [file 41419_2020_2468_MOESM4_ESM.tif]

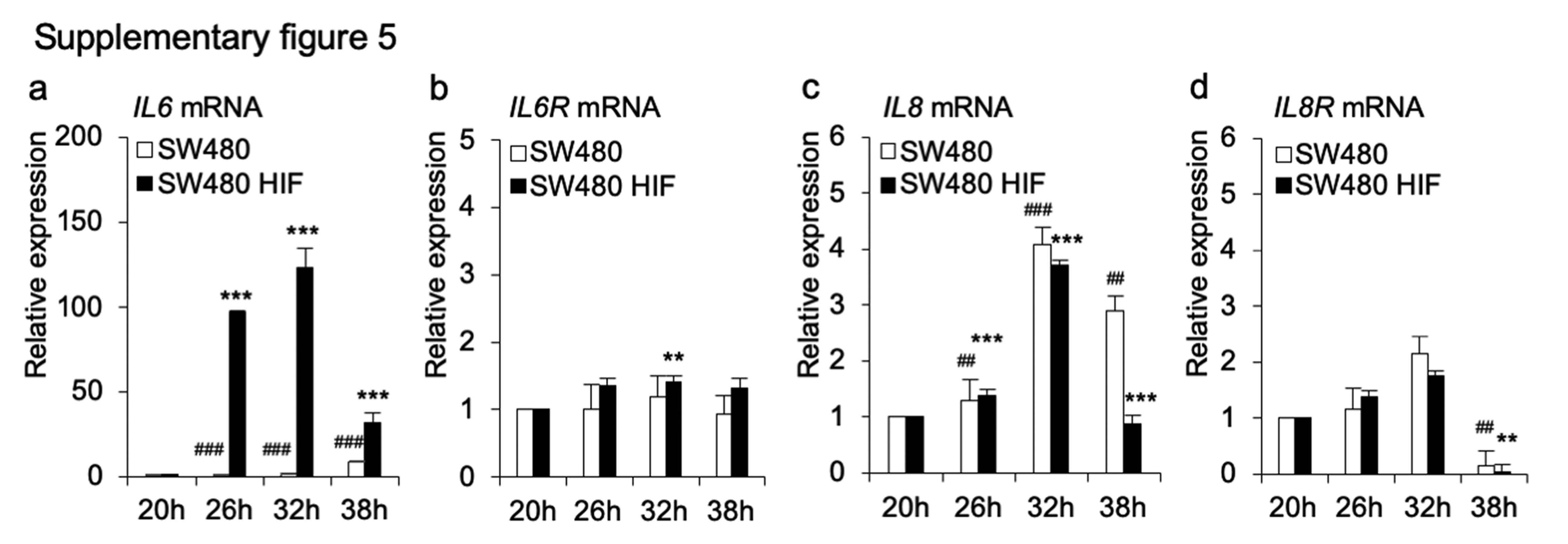

Supplement: Supplementary file 5 — Supplementary Figure 5. [file 41419_2020_2468_MOESM5_ESM.tif]

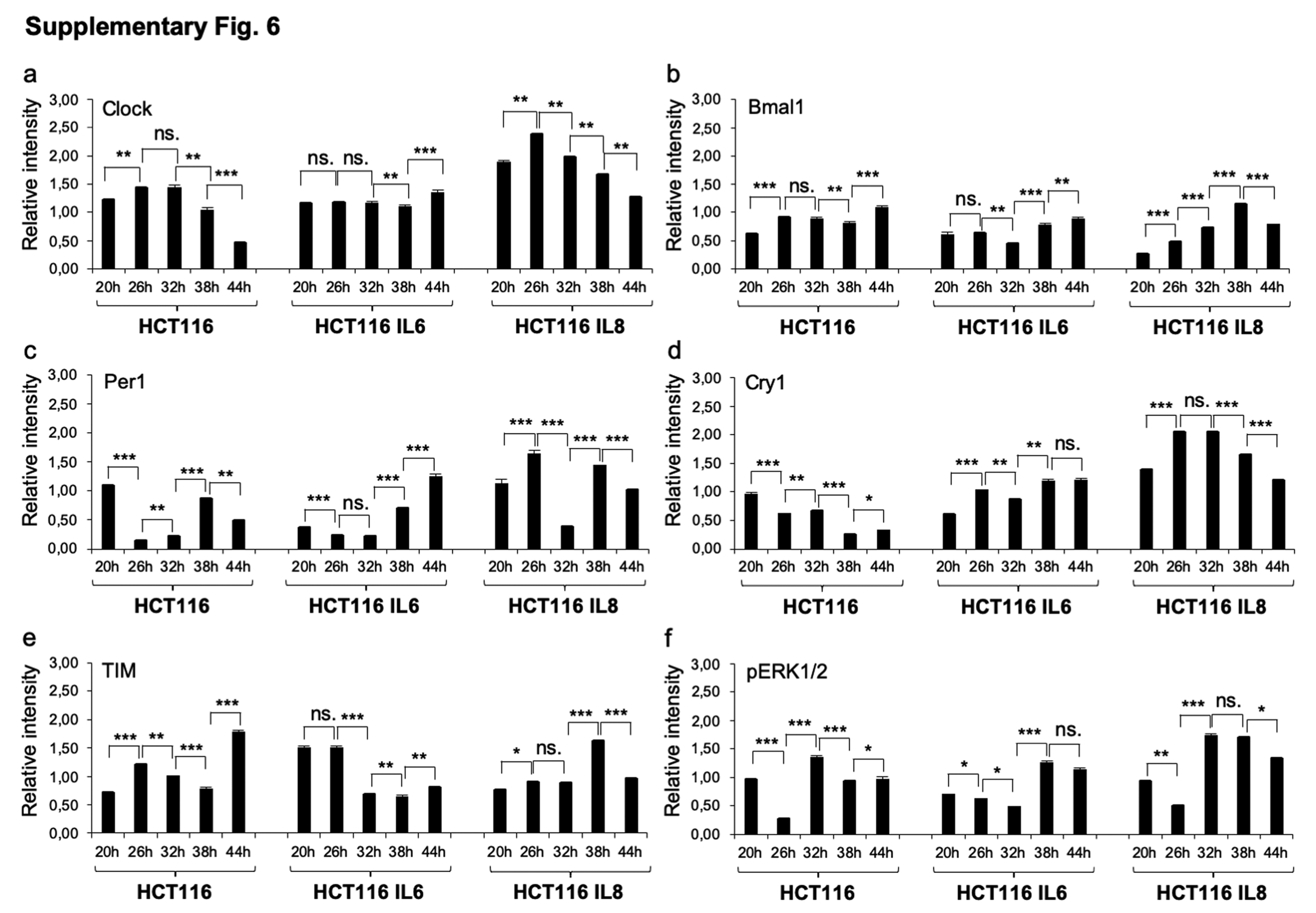

Supplement: Supplementary file 6 — Supplementary Figure 6. [file 41419_2020_2468_MOESM6_ESM.tif]
