# Supplementary material for: Effect of naive and cancer-educated fibroblasts on colon cancer cell circadian growth rhythm
Source: Cell Death Dis. 2020 Apr 27;11(4):289. doi: 10.1038/s41419-020-2468-2 (PMC7184765; doi:10.1038/s41419-020-2468-2)
Supplement: Supplementary file 8 — SUPPLEMENTARY FIGURE AND TABLE LEGENDS [file 41419_2020_2468_MOESM8_ESM.docx]

**SUPPLEMENTARY FIGURE AND TABLE LEGENDS**

**Supplementary Fig. 1.** **The analysis of mRNA expression of markers that characterize cancer-associated fibroblasts (CAFs)**. **a.** *Tenascin* and *fibroblast activation protein* (*FAP*) mRNA expression were both significantly (p<0.001) downregulated in fibroblasts isolated from tumours, suggesting that fibroblasts isolated from colon adenocarcinoma have not differentiated to CAFs and are therefore called in this work as tumour associated fibroblasts (TAFs). **b**-**d.** Time-dependent expression of *Tenascin, FAP*, and *Collagen 1A1* (*Col1A1*) in naïve fibroblasts and in TAF support the data shown in panel **a**. The p-values (*p < 0.05, **p < 0.01, and ***p < 0.001) in panel **a.** were calculated by comparing naïve fibroblasts to TAF and in panels **b**-**d.** by comparing the timepoints 20h to 26h, 26h to 32h, 32h to 38h, and 38h to 44h in naïve fibroblast and in TAF samples.

**Supplementary Fig. 2. Western blot intensity values for naïve fibroblasts, TAFs, HCT116 cells, HCT116 cells co-cultured with naïve fibroblasts, and HCT116 cells co-cultured with TAFs.** The p-values are *p < 0.05, **p < 0.01, and ***p < 0.001.

**Supplementary Fig. 3. The expression analysis of clock genes in SW480, HIF, and in the co-culture.** The mRNA expression of **a.** *Clock*, **b.** *Bmal1*, **c*.*** *Per1*, **d**. *Cry1*, and **e.** *TIM* was analysed in SW480 cells cultured alone, in primary HIF single cell cultures, and in SW480 cells cultured together with primary HIF. The p-values (*p < 0.05, **p < 0.01, and ***p < 0.001) were calculated by comparing the timepoints 20h to 26h, 26h to 32h, and 32h to 38h in each cell type.

**Supplementary Fig. 4. Array data showing the protein expression in naïve fibroblasts and in TAFs.**

**Supplementary Fig. 5.** **The expression analysis of cytokines and their receptors in SW480 colon cancer cells cultured alone or at the presence of primary HIF.** The mRNA expression of **a.** IL6, **b.** IL6R, **c.** IL8, and **d.** IL8R. The p-values (*p < 0.05, **p < 0.01, and ***p < 0.001) were calculated by comparing the timepoints 20h to 26h, 26h to 32h, and 32h to 38h in each cell type.

**Supplementary Fig. 6. Western blot intensity values for HCT116 cells treated with IL6 and IL8.** The p-values are *p < 0.05, **p < 0.01, and ***p < 0.001.

**Supplementary Table 1. Statistical significance for the array data showing the protein expression in naïve fibroblasts and in TAFs.** **a,b.** The p-values for the relative intensity values shown in the supplementary figure 4a. **c**,**d.** The p-values for the relative intensity values shown in the supplementary figure 4b. **e**,**f.** The p-values for the relative intensity values shown in the supplementary figure 4c.
